# Hepatic hydatid cyst ruptured from the transdiaphragmatic path to the pleural cavity

**DOI:** 10.1590/0037-8682-0610-2022

**Published:** 2023-02-20

**Authors:** Ramazan Orkun Önder, Serdar Aslan

**Affiliations:** 1 Giresun University, Faculty of Medicine, Department of Radiology, Giresun, Turkey.

**FIGURE 1:**
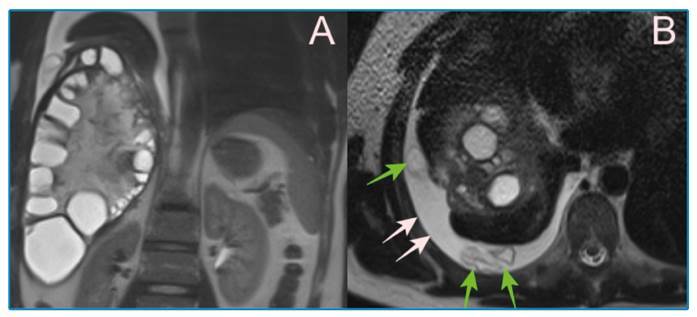
CE-3b type (daughter cysts within a solid matrix) cystic lesion in the dome of the liver **(A)**, pleural effusion in the right hemithorax (white arrows) and membranous daughter cysts inside (green arrows) observed on MRCP **(B)**.

**FIGURE 2:**
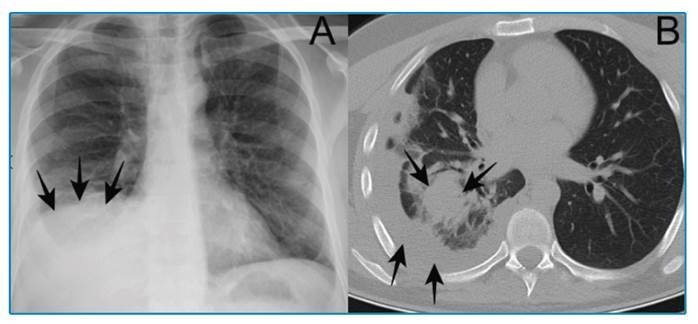
Consolidation and pleural effusion in the right inferior segment of the lung as seen on posteroanterior radiograph examination **(A)** and thorax CT **(B)**.

A 40-year-old man was admitted to our emergency department complaining of weakness, pain in the right hemithorax, and shortness of breath. The patient’s medical history was unremarkable. Physical examination revealed tenderness in the upper right quadrant on palpation. Laboratory tests showed elevated white blood cells (16.5x10⁹ /L), aspartate aminotransferase (145 U/L), alanine aminotransferase (163 U/L), direct bilirubin (1.71 mg/dL), and total bilirubin (1.97 mg/dL). Abdominal ultrasonography (US) revealed a CE-3b type cystic lesion in the dome of the liver. Magnetic resonance cholangiopancreatography (MRCP) showed pleural effusion in the right hemithorax and membranous daughter cysts in addition to the findings noted in US (Figure 1). Posteroanterior radiography and thoracic computed tomography (CT) revealed consolidation and pleural effusion in the right inferior segment of the lung (Figure 2). Thoracic and general surgeons planned to perform surgery for the patient after antibiotic therapy (ceftriaxone 2 g/day and clarithromycin 500 mg/day) and albendazole (800 mg/day) treatment. Hydatid cyst ruptures can be spontaneous, iatrogenic, or traumatic. Ruptured hydatid cysts may present with asymptomatic or anaphylactic reactions. Coughing, dyspnea, fever, hemoptysis, flank pain, chest pain, and secondary pneumonia with pressure to the neighboring bronchi can develop due to pulmonary rupture of the hydatid cyst on the diaphragmatic surface of the liver^1^ ^-^ ^3^. Although rare, primary lesions at the dome of the liver can rupture into the lungs through the diaphragmatic route. Atypical forms of the disease should be considered as differential diagnoses in regions with a high prevalence of echinococcosis.
